# Electrical Brain Activity and Its Functional Connectivity in the Physical Execution of Modern Jazz Dance

**DOI:** 10.3389/fpsyg.2020.586076

**Published:** 2020-12-15

**Authors:** Johanna Wind, Fabian Horst, Nikolas Rizzi, Alexander John, Wolfgang I. Schöllhorn

**Affiliations:** Training and Movement Science, Institute of Sport Science, Johannes Gutenberg-University Mainz, Mainz, Germany

**Keywords:** EEG, dance, physical activity, rhythm, coherence, connectivity, brain activity, power spectrum

## Abstract

Besides the pure pleasure of watching a dance performance, dance as a whole-body movement is becoming increasingly popular for health-related interventions. However, the science-based evidence for improvements in health or well-being through dance is still ambiguous and little is known about the underlying neurophysiological mechanisms. This may be partly related to the fact that previous studies mostly examined the neurophysiological effects of imagination and observation of dance rather than the physical execution itself. The objective of this pilot study was to investigate acute effects of a physically executed dance with its different components (recalling the choreography and physical activity to music) on the electrical brain activity and its functional connectivity using electroencephalographic (EEG) analysis. Eleven dance-inexperienced female participants first learned a Modern Jazz Dance (MJD) choreography over three weeks (1 h sessions per week). Afterwards, the acute effects on the EEG brain activity were compared between four different test conditions: physically executing the MJD choreography with music, physically executing the choreography without music, imaging the choreography with music, and imaging the choreography without music. Every participant passed each test condition in a randomized order within a single day. EEG rest-measurements were conducted before and after each test condition. Considering time effects the *physically executed dance without music* revealed in brain activity analysis most increases in alpha frequency and in functional connectivity analysis in all frequency bands. In comparison, *physically executed dance with music* as well as *imagined dance with music* led to fewer increases and *imagined dance without music* provoked noteworthy brain activity and connectivity decreases at all frequency bands. Differences between the test conditions were found in alpha and beta frequency between *the physically executed dance* and *the imagined dance without music* as well as between the *physically executed dance with* and *without music* in the alpha frequency. The study highlights different effects of a physically executed dance compared to an imagined dance on many brain areas for all measured frequency bands. These findings provide first insights into the still widely unexplored field of neurological effects of dance and encourages further research in this direction.

## Introduction

As cave paintings indicate, the human ability to dance is an inherent activity of humans, since these paintings show whole-body movements as they are used in dance (Appenzeller, 1998; Ward, 2002; Bramble and Lieberman, 2004). Nowadays, the forms of expression of dance differ in numerous categories or styles, such as classical dance, modern dance, folk dance, spiritual dance. What most of these dance categories have in common is that there is a movement of the whole body to music (Brown et al., 2006). Besides the pleasure of watching dancers at theater performances or sport competitions, dance receives a growing interest for health reasons. Recent studies indicate a supportive effect of dance in conjunction with medical therapies for breast cancer (Dibbell-Hope, 2000; Sandel et al., 2005), diabetes (Murrock et al., 2009), fall prevention (Franco et al., 2016; Merom et al., 2016), dementia (Guzmán-García et al., 2013; Barnes et al., 2015), or brain plasticity (Rehfeld et al., 2018). An augmentation of the impact could be established when implementing dance therapy in the treatments for autism (Koch et al., 2015), depression (Jeong et al., 2005; Haboush et al., 2006; Koch et al., 2007) and Parkinson Disease (Hackney and Earhart, 2009, 2010; Duncan and Earhart, 2012; Houston and McGill, 2013). In its multi-faceted ways, dance is assumed to promote human creativity (Fink and Woschnjak, 2011), social competence (Lobo and Winsler, 2006), enjoyment (Gao et al., 2013a,b), contributes to well-being (Mansfield et al., 2018) and can lead to the experience of flow (Bernardi et al., 2018).

The science-based evidence for dance-associated improvements of the health or well-being of humans is ambiguous due to heterogeneous study designs, populations, intervention protocols, dance categories as well as types and severities of fields of application. Consequently, there is little consensus on how dance interventions should be designed to achieve optimal effects for the specific needs of an individual person. Understanding the factors of dance that affect neuronal responses to induce neuroplastic changes (Hotting and Roder, 2013) can help to optimize interventions through individualized and targeted dance interventions.

While it has been shown that several years of dance training led to an increased brain activity in alpha and beta frequencies (Ermutlu et al., 2015), at premotor and parietal brain areas (Cross et al., 2009) as well as in increased gray matter volume (Müller et al., 2017), the underlying neurophysiological mechanisms leading to neuroplastic changes in experienced dancers are insufficiently understood (Schwender et al., 2018).

Research investigating the neuronal processes of observation and imagination of dance can provide first insights in this regard. EEG-studies examining brain activity of dance experts showed that the audio-visual observation of dance was accompanied by increased brain activity in theta frequency at the frontocentral area and a decreased brain activity in alpha frequency (Poikonen et al., 2018a) and lower beta frequency bands (Orgs et al., 2008). In laymen, dance observation led to increased functional connectivity in theta between frontocentral and frontopolar brain areas and in gamma frequency between central and frontopolar ones (Poikonen et al., 2018b). Brain activity analysis during the imagination of the own dance choreography increased the right hemispheric alpha-frequencies (Fink et al., 2009).

To the best of our knowledge, only one study has so far investigated the acute effects of the physical execution of dance on the brain using EEG (Cruz-Garza et al., 2014). The study showed an increase in activation at premotor, motor, and parietal areas. Because of the multi-faceted character of dance with its very diverse and simultaneous stimuli (visual, acoustic, proprioceptive) and parallel activities (arm, trunk, leg and head movements), analyzing functional connectivity (for definition see Bowyer, 2016) might provide further insights into the interaction of specific brain regions that are specialized of processing dance movements. Thus, an analysis of brain power and functional connectivity may lead to a more detailed understanding of the acute effects of dance on the brain network.

Additionally, there remain several aspects of the acute effects on electromagnetic brain activity and functional connectivity after a physically executed dance, about which relatively little is known. In particular, the different facets of dance, such as music, movement in space, as well as the combination of music accompanied dance were not investigated so far. For this reason, this pilot study examines acute effects of a physically executed Modern Jazz Dance choreography with and without music and compared it to the imagination of this choreography with and without music, by means of brain activity and functional connectivity analysis. We hypothesize, that the physically executed Modern Jazz Dance leads to a higher electromagnetic brain activity increase and cooperation between brain areas than the pure imagination of the exact same dance. This suggestion is based on the one hand on functional connectivity studies, which have been carried out with fine-motor finger tapping tasks and resulted in an increased connectivity (Rappelsberger et al., 1994; Classen et al., 1998; Gerloff et al., 1998; Manganotti et al., 1998). Since we investigate the gross-motor activity dance, which is barely examined in EEG studies in its physically executed form, we present on the other hand studies which analyzed other gross-motor activities like running or cycling. Effects immediately after gross-motor activity resulted in an intra-hemispheric lower alpha coherence in bilateral parietal-frontal and parietal-central areas, in a bilateral parietal-frontal coherence in upper alpha band (Babiloni et al., 2011) as well as in a coherence increase between frontoparietal network and frontal cortex (Raichlen et al., 2016). Studies investigating connectivity during gross-motor activity (e.g., cycling) showed increased connectivity over the whole scalp in all frequency bands (di Fronso et al., 2018) and in a single-subject study in alpha frequency across all brain areas when focus of attention was kept external (Comani et al., 2013). Concerning brain activity, acute effects in gross-motor activity like running led to an increased alpha activity in left frontal regions (Schneider et al., 2010) and after the execution of cycling to an increased alpha frequency in parietal regions (Schneider et al., 2009).

Furthermore, we hypothesize differences between the conditions with and without music, since listening to music led to alterations in brain activity in earlier investigations. Studies investigating passive music listening were accompanied with increased activation in auditory cortex (Kristeva et al., 2003), in frontal midline theta power (Sammler et al., 2007) as well as in right frontal and temporal regions in beta and alpha frequencies (O’Kelly et al., 2013). Even an enhanced activation in motor cortex could be explored (Kristeva et al., 2003; Callan et al., 2006; Gordon et al., 2018), which supports the idea that motor planning processes are involved in music perception. Next to passive music listening, music listening while exercising (running) led on the one hand to an up-regulation of beta waves in frontal and prefrontal-central regions (Bigliassi et al., 2018). On the other hand, power of low-frequency waves, like theta, at frontal, central and parietal regions were down-regulated. However, this effect was not seen while listening to music at rest (Bigliassi et al., 2016).

Through this exploratory analysis, we assume to enlighten if and to what extent the type of movement, the music or the combination of music and movement are relevant for effects of dance interventions that could be observed in several health-related studies.

## Materials and Methods

### Participants

Eleven female participants with a mean age of 24.3 years (*SD* = 2.45; range 21–29) volunteered for this pilot study. Starting from the aspect that no information about the probability of the hypotheses is provided and no generalization of hypothesis can be made with the underlying type of study designs regardless of the sample size, the sample size seems to be sufficient to provide reasonable indications for future studies according to Fisher’s original statistics (Fisher, 1955; Stegmüller, 1973; Gigerenzer, 2004; Nuzzo, 2014). Participants without any dance training experience were recruited within the Johannes Gutenberg-University Mainz. All participants were healthy, free from neurological diseases, and right-handed. The participants were informed about the experimental procedure and provided written informed consent prior to their participation. The design and conduct of the study were in accordance with the general principles outlined in the Declaration of Helsinki and the local ethics committee of the Johannes Gutenberg-University of Mainz (Germany) approved the study.

### Experimental Procedure

**FIGURE 1:**
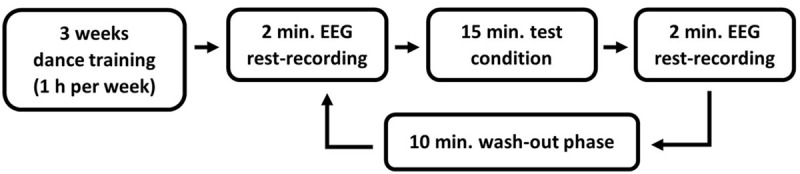
Overview of the experimental procedure.

Before the test started, all participants attended a three-week training course in Modern Jazz Dance with a one-hour session per week in the sports facility of the Johannes Gutenberg-University Mainz. In this course a given sequence of dance moves was taught. All training courses were the same for all participants. In order to look for effects that can possibly be assigned to findings of behavioral studies with health effects, a within subject design with multiple pre- posttest designs was chosen. Three to five days after the last training session, EEG measurements were taken (see Figure 1 for an overview of the entire experimental procedure).

The EEG measurements took place in a dimly lit room, in which all test conditions were conducted. Each test condition was preceded and followed by a 2-min resting condition that was used for the measurement of the brain activity using EEG before and after each test condition. An eyes-open resting condition was chosen to avoid the increase of alpha waves due to the closing of the eyes (Barry and Blasio, 2017). A wash-out phase of 10 min took place between the individual test conditions (Bailey et al., 2008). During the resting and wash-out phases the participants were asked to sit calmly on a chair facing a white wall.

All participants accomplished each test condition for the Modern Jazz Dance (MJD) choreography in a within-subject design. Four test conditions had to be passed in a randomized order with opened eyes: (1) dance the MJD physically with music, (2) executing the same dance physically without music, (3) imagining the dance with music, as well as (4) imagining the dance without music.

### EEG Data Acquisition

The EEG measurements were recorded with the Micromed SD LTM 32 BS amplifier (Venice, Italy) and the System Evolution Plus Software (Venice, Italy). Nineteen electrodes (Fp1, Fp2, F3, F7, Fz, F4, F8, C3, Cz, C4, T3, T4, P3, T5, Pz, P4, T6, O1, O2) were applied according to the international 10–20-system with the reference electrode attached to the nose. The electrode impedances were kept below 10 kΩ. The EEG signals were digitized at a sampling rate of 1024 Hz. The electrooculography (EOG) was affixed at the lateral orbital and the medial upper rim of the right eye.

### EEG Data Preprocessing

For EEG data preprocessing a bandpass filter was set to 2–80 Hz and spectrum interpolation (Leske and Dalal, 2019) was used to eliminate frequency peaks at 50 Hz. Additionally, an Independent Component Analysis (ICA) was computed by means of the Matlab-based EEGLAB-toolbox 2019 (Delorme and Makeig, 2004). Interference-prone channels and recurrent movement artifacts, like eye blinks or sweat artifacts, were deducted from the signal.

### EEG Data Analysis

The measurements of the electrical brain activity were analyzed by means of the same EEGLAB-toolbox as mentioned in EEG Data Preprocessing (MathWorks, United States; Swartz Center of Computational Neuroscience, San Diego, CA, United States) (Delorme and Makeig, 2004). Functional connectivity analyses COH and ICOH were conducted by means of the Matlab-based METH-toolbox (MEG and EEG Toolbox of Hamburg) (Dept. of Neurophysiology and Pathophysiology; University Medical Center Hamburg-Eppendorf).

#### EEG Power Spectrum

The power spectrum was computed by using the Fast-Fourier-Transformation with a Hamming window, a window size of 2048 samples (2 s) and a window-overlap of 50%. The mean power spectra were calculated for the theta (3.5–7.5 Hz), alpha (7.5–12.5 Hz), beta (12.5–30 Hz) and gamma (30–70 Hz) frequency bands. These frequency ranges were progressed by Zschocke and Hansen (2012). The power spectrum was analyzed for each test condition (*physically executed dance with music, physically executed dance without music, imagined dance with music, imagined dance without music*), frequency band (theta, alpha, beta, gamma), and electrode (Fp1, Fp2, F3, F7, Fz, F4, F8, C3, Cz, C4, T3, T4, P3, T5, Pz, P4, T6, O1, O2).

#### EEG Functional Connectivity

Functional connectivity was analyzed between all 19 electrodes using the magnitude-squared coherence (COH) (Shaw, 1984; Comani et al., 2013; Wang et al., 2015; di Fronso et al., 2018; Xiao et al., 2018) and the Imaginary Part of Coherency (ICOH) (Nolte et al., 2004; Garcia Dominguez et al., 2013; Hohlefeld et al., 2013; Jamieson and Burgess, 2014; Lord and Opacka-Juffry, 2016). COH describes the phase stability of two oscillating signals over a period of time (Guevara and Corsi-Cabrera, 1996). The main disadvantage of this measure is a possible influence of volume conduction, which is understood as the reflection of a single source in multiple electrodes (Fein et al., 1988) and could provoke an inflation of coherence in those electrodes. Since volume conduction is assumed to lead to zero-phase lagged signals, the ICOH might be a measurement of functional connectivity that claims to be less sensitive to the assumed model of volume conduction. ICOH tries to bypass the issue of volume conduction by considering only time-lagged signal parts, respectively, by eliminating coherence caused by instantaneous activity (Nolte et al., 2004). However, ICOH is a rigorous measure of coherence, because not all zero-phase lagged signal parts might be caused by volume conduction. Similarly, if the signal source for volume conduction is not assumed to be centrally located then volume conduction can lead to relative phase lags too. We therefore analyzed both, COH (which may overestimate the connectivity due to volume conduction) and ICOH (which may underestimate the actual connectivity because of excluding all zero-phase lagged signal parts). In order to compare both coherence measures, we use the absolute value of ICOH. Accordingly, COH and ICOH are indexes of connectivity which range from 0 to 1. In the case of COH 0 means no interaction and 1 complete or ideal interaction of two EEG electrodes and with ICOH analysis we certainly can say that there is true brain interaction.

Coherency was calculated with following formula:

(1) $$C_{i\,j}\,\left(f\right)=\frac{S_{i\,j}\,\left(f\right)}{{\left(S_{i\,i}\,\left(f\right)\,S_{j\,j}\,\left(f\right)\right)}^{1\,/\,2}}$$

The cross-spectrum (*S_*ij*_(f)*) and the power-spectrum (*S_*ii*_(f)* and *S_*jj*_(f)*) were estimated by Welch’s method with a Hanning window, a window-size of 4096 samples (4 s) and an overlap of 50% for the following frequencies: theta (3.5–7.5 Hz), alpha (7.5–12.5 Hz), beta (12.5–30 Hz) and gamma (30–70 Hz) frequency band (Zschocke and Hansen, 2012). According to the applied 19 electrodes, each type of connectivity analysis resulted in 171 electrode pairs.

#### EEG Statistical Analysis

The pre-processed data of the power and connectivity analysis were transferred to the SPSS-software (IBM, SPSS 23) for statistical evaluation.

The acute test condition effect, in other words time alterations, were analyzed for each test condition, frequency band and electrode resp. electrode pair, using a Wilcoxon-Test for non-normally distributed data. In order to determine differences between the test conditions, difference values (subtraction of pre-rest-measurement from post-rest-measurement) were first calculated of the test conditions. Statistical differences between the difference-values of the test conditions were analyzed by means of a Friedman-Test and Dunn-Bonferroni corrected post-hoc tests. According to the Fisher statistics, the statistical significance was set at p-value ≤ 0.05. Additionally, the effect size, mainly assigned to the Neyman and Pearson (1933) statistics was converted to Cohen’s *r* (1992) with the following effect sizes: *r* = 0.1 (small effect), *r* = 0.3 (medium effect), *r* = 0.5 (large effect).

Since the power analysis compares 19 electrodes and the connectivity calculation includes 171 electrode pairs per frequency band and condition, the new significance level calculated with an FDR-correction (False Discovery Rate) would be in case of time alteration analysis in connectivity *p* ≤ 0.0003 and in power spectrum *p* ≤ 0.0026. Due to the rigorous ICOH analysis and the high alpha correction, we focus on statistically significant time alterations with an alpha threshold of *p* ≤ 0.05, with high effect sizes (*r* ≥ 0.5). Based on the problem of volume conduction using COH and the underestimate of possible connectivity by means of ICOH, only results of electrode pairs are interpreted, which are statistically significant with large effect size in time alterations and with all ranges of effect size between test conditions, in both connectivity measurements (COH and ICOH). This eliminates the influence of volume conduction with the help of the ICOH, and COH allows the unambiguous interpretation of a reduced or increased interaction.

## Results

### Power Spectrum Results

Table 1 presents the descriptive and inferential statistical values of statistically significant time effects with high effect sizes (*r* ≥ 0.5), from pre- to post-rest-measurement.

**TABLE 1:** Significant time effects of power spectrum.

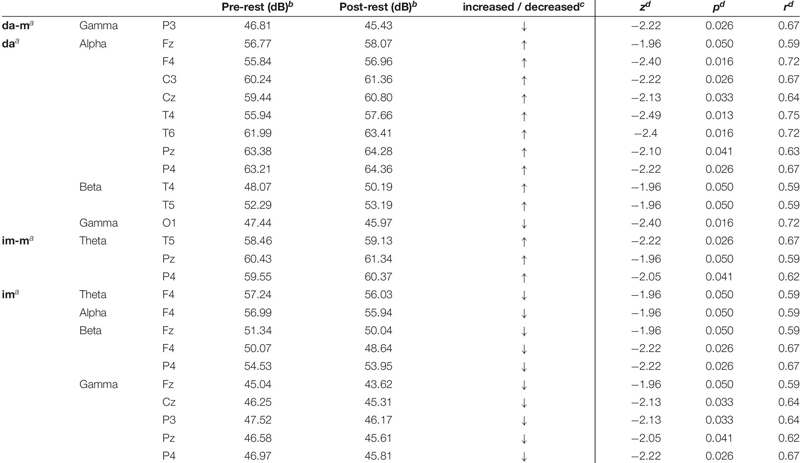

*Statistically significant time effects of brain activity spectrum. Left column descriptive values, right column – statistical values. ^*a*^da-m, physically executed dance with music; da, physically executed dance without music; im-m, imagined dance with music; im, imagined dance without music. ^*b*^pre- and post-rest values in decibel. ^*c*^arrow up, brain activity increased from pre- to post-rest measurement; arrow down, brain activity decreased from pre- to post-rest measurement. ^*d*^*z*-value of the Wilcoxon-test, *p*-value, *r*-value of the effect size.*

Significant time effects were obtained for the *physically executed dance with music* in gamma frequency with a decreased brain activity in the parietal lobe (electrode P3). The *physically executed dance without music* revealed an increase frontal (Fz, F4), central (C3, Cz), temporal (T4, T6) and parietal (Pz, P4) in alpha frequency as well as temporal (T4, T5) in beta frequency and furthermore a decrease occipital (O1) in gamma frequency. The *imagined dance with music* showed an increase temporal (T5) and parietal (Pz, P4) in theta frequency. The *imagined dance without music* revealed in all frequency bands decreases in brain activity. Reductions in theta and alpha frequency in frontal (F4) lobe, in beta frequency frontal (Fz, F4) as well as parietal (P4) and in gamma frequency in the frontal (Fz), central (Cz) and parietal (Pz, P4) lobe have been identified.

Table 2 shows the descriptive and inferential statistical values of statistically significant differences between the four test conditions.

**TABLE 2:** Significant differences between test conditions of power spectrum.

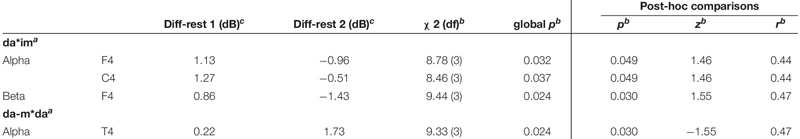

*Statistically significant differences between test conditions with presentation of the Diff-rest values. Left column descriptive values, right column – statistical values. ^*a*^statistical differences between test condition pairs, da-m, physically executed dance with music; da, physically executed dance without music; im, imagined dance without music. ^*b*^ χ2 with degrees of freedom of Friedman-test, *r*-value effect size of the Friedman-test, z-value of the Friedman-test. ^*c*^Diff-rest 1, difference value from pre- to post-rest-measurement of the left test condition from test condition pair; Diff-rest 2, difference value from pre- to post-rest-measurement of the right test condition from test condition pair.*

Significant differences were revealed in alpha frequency band at the frontal (F4) and central (C4) brain lobe as well as in beta frequency band at frontal lobe (F4) between the *physically executed dance without music* and the *imagined dance without music*. At all electrodes activation increased in the *physically executed dance without music* and decreased in the *imagined dance without music*. Furthermore, alpha power significantly differed at the temporal lobe (T4) between the *physically executed dance with music* and the *physically executed dance without music* with a higher increased activity in the *physically executed dance without music*.

### ICOH and COH Results

**FIGURE 2:**
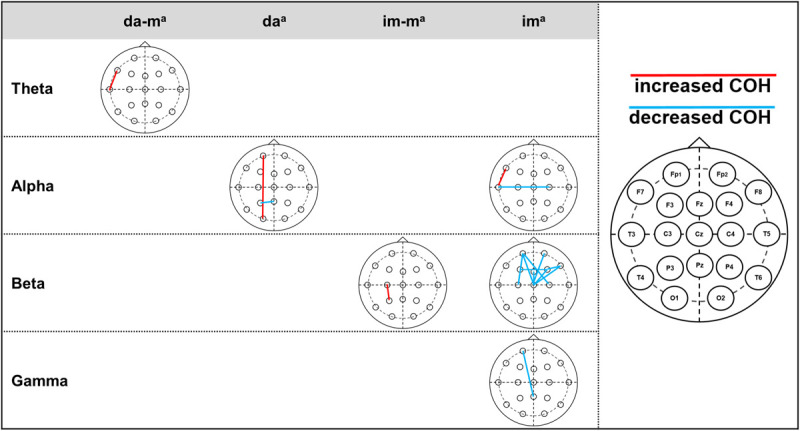
Significant time effects for jointly electrode pairs of COH and ICOH. Statistically significant time effects of the jointly electrode pairs of COH and ICOH with presentation of the increased or decreased COH. Right column: head model of 10–20 system, electrode pairs with increased COH presented by red lines, electrode pairs with decreased COH presented by blue lines. ^*a*^da-m, physically executed dance with music; da, physically executed dance without music; im-m, imagined dance with music; im, imagined dance without music.

Table 3 and Figure 2 present the descriptive and inferential statistical values of electrode pairs with statistically significant time effects with large effect size, which occur in COH and ICOH in parallel. The *physically executed dance with music* led to statistically significant increase in theta frequency at electrode pair F7-T3. For the *physically executed dance without music* an increase could be observed in alpha frequency between electrode pairs of Fp1-O1 and a decrease for P3-Pz. The *imagined dance with music* led to an increased connectivity between C3-P3 in beta frequency. Most alterations over time were revealed in the *imagined dance without music* with an increased connectivity at F7-T3 and a decreased in T3-C4 at alpha frequency. Beta frequency decreases were provoked between electrode pairs Fp1-C3, Fp1-Cz, Fp1-C4, Fp2-Cz, F3-F4, Fz-Cz, F4-F8, F4-Cz, F8-Cz and in gamma frequency between Fp1-Pz. See Tables 4 and 5 in Supplementary Material for significant time effects in the four test conditions, separated for COH and ICOH analysis.

**TABLE 3:** Significant time effects for electrode pairs of both connectivity analysis (COH and ICOH).

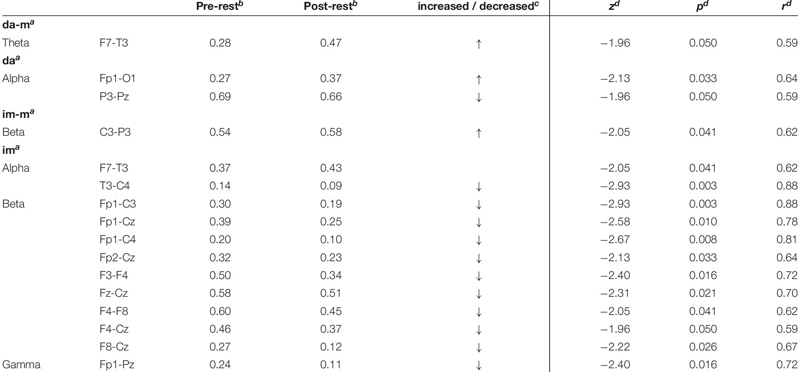

*Statistically significant time effects of the electrode pairs of both connectivity measurements (COH and ICOH) with presentation of the COH values. Left column descriptive values, right column – statistical values. ^*a*^da-m, physically executed dance with music; da, physically executed dance without music; im-m, imagined dance with music; im, imagined dance without music. ^*b*^pre- and post-rest values of COH. ^*c*^arrow up, connectivity increased from pre- to post-rest measurement; arrow down, connectivity decreased from pre- to post-rest measurement. ^*d*^z-value of the Wilcoxon-test, *p*-value, *r*-value of the effect size.*

See Tables 6 and 7 in Supplementary Material demonstrate the descriptive and inferential statistical values of electrode pairs with statistically significant differences between test conditions, which occur separated in COH (Table 6 in Supplementary Material) and ICOH (Table 7 in Supplementary Material). Solely the electrode pair Fp1-F4 in gamma frequency showed in both analysis (COH and ICOH) differences between the *physically executed dance without music* and *imagined dance without music* with an increased connectivity in the *physically executed dance without music*.

## Discussion

The current study compared the acute neurophysiological effects between a physically executed and imagined dance of a MJD choreography. Increased brain activity and functional connectivity were found after a physically executed dance with and without music. In comparison, the brain activity and functional connectivity increased after imagination with music but decreased after imagination without music. Our results indicate differences between a physically executed and imagined dance, thus closing initial research deficits of neurophysiological effects of a physically executed dance. The findings underline the need to conduct studies on physically executed dance in order to further understand and optimize targeted dance interventions. However, due to the small sample size of eleven female participants, this study can be categorized as a pilot study, therefore, no claim can be made to generalizability of the results.

### Impact of Physically Executed Dance With Music

The *physically executed dance with music* led merely to alterations in parietal cortex (P3) with decreased brain activity in gamma frequency. This is an atypical phenomenon regarding to the usual features connected to parietal lobe and gamma frequency. Both, an increased brain activity in parietal lobe and an increased gamma frequency are cues for multisensory and sensomotoric integration (Singer and Gray, 1995) as well as for guidance and control of movements (Kolb and Ian, 2015). If this is a stable pattern in *physically executed dance with music*, it can be explained as a process of brain-sparing power due to an automatically and solely performed choreography. Furthermore, there was no need to react spontaneously to an eventually changing environment, as the participants were familiar with the premises, which might provoke an increase in parietal activity. Whether dance together with other persons would lead to a parietal increase in brain activity due to visual attention of the co-dancers, has to be clarified in further studies.

In difference to the decreased brain activity, functional connectivity increased in theta frequency between frontal and temporal brain lobe (F7-T3), which could indicate towards the involvement of the working memory, attentional (temporal cortex) and awareness processing (frontal cortex) (Chayer and Freedman, 2001; Malik and Amin, 2017), due to the parallel recognition of the rehearsed choreography, the coordination of the body in space as well as to music and at least for the accurate movement execution. The involvement of frontal lobe could be furthermore assigned to executive functions (Zschocke and Hansen, 2012; Bähr and Frotscher, 2014). The latter are associated i.a. with processes of concentration, self-control, working memory and cognitive flexibility (Diamond, 2013), which are required while dancing. The impact of dance in promoting executive functions needs further research, however, current findings give indications and show a possibly beneficial application of dance, for example in educational establishments. An interesting point of view is the finding of Sauseng et al. (2005), who showed an increase in frontoparietal network after a visuospatial working memory task, which is associated with central executive functions (Sauseng et al., 2005). Central executive functions interact with the phonological loop, the visual-spatial system, and controls the subordinated processes, to make latter as efficient as possible (Swanson, 2015). This is also demanded in dance because of the decision when, where and how to coordinate the limbs in time and in space. Whether the increased theta frequency after *physically executed dance with music* influence healing processes (Jeong et al., 2005; Haboush et al., 2006; Koch et al., 2007; Hackney and Earhart, 2009, 2010; Duncan and Earhart, 2012; Houston and McGill, 2013; Koch et al., 2015) in the form of increased dopamine production (Kjaer et al., 2002) or by activating the parasympathetic system (Takahashi et al., 2005) or supporting the absorption of nutrients (Mourad and Saade, 2011) needs to be investigated in more detail.

### Impact of Physically Executed Dance Without Music

The observed findings in brain activity in the alpha and beta frequency in the *physically executed dance without music* can be explained plausibly by the requirements while dancing, like perceiving the room, planning dance steps, executing dance movements precisely and coordinating limbs in the required manner in space. *Physically executed dance without music* led presumably to memory and attentive processes (Klimesch, 1997; Hanslmayr et al., 2011) due to an increased alpha frequency in the frontal (Fz, F4), central (C3, Cz), temporal (T4, T6) and parietal (Pz, P4) cortex, since these processes are needed for sensory and planning functions (feature of frontal and temporal lobe) (Zschocke and Hansen, 2012; Kolb and Ian, 2015) while dancing. An increased brain activity in central and parietal cortex occur typically in motoric processes due to its characteristic of processing and controlling limb movements (central lobe) as well as movement guidance or spatial cognition (parietal lobe) (Kolb and Ian, 2015). Based on the simultaneous increment of alpha in frontal cortex, which represents relaxed wakefulness, it could be speculated that dance leads to deep concentration (Cantero et al., 2002). Increased frontal alpha frequencies were already observed during the imagination of dance (Fink et al., 2009) and after several years of dance experience (Ermutlu et al., 2015). The present study revealed similar brain patterns, however, immediately after a physically executed dance with gross-motor movements.

An increased beta activity emerges usually during voluntary movements and more often after motoric activities (Engel and Fries, 2010). In connection to the temporal lobe (T4, T5) it might be a clue for conscious attention, the transformation of sensory input into motoric output or the emotions caused by expressive dance (Thangavel et al., 2008; Adair and Meador, 2014; Simons and Johnsrude, 2014; Gonzalez and Flindall, 2015). The decreased occipital (O1) brain activity in gamma frequency as well as the decreased connectivity between parietal electrodes (P3-Pz) in alpha frequency lead to the same assumption as in *physically executed dance with music* with decreased parietal gamma frequency. These decreases could be a clue for brain-sparing power or the reduced demand in visual processing due to the automatically executed solo dance.

Increased connectivity in alpha frequency between the frontal and occipital lobe (Fp1-O1) confirm the findings in brain activity. Prefrontal cortex represents the selection and the control of movements at the right place and time (Kolb and Ian, 2015). In connection to the occipital lobe, with its characteristics of visual processing, visuomotor guidance and body analysis (Adair and Meador, 2014; Carter, 2014; Kolb and Ian, 2015; Galetta, 2017), it reflects important processes while dancing. The simultaneous appearance of anterior (frontal) and posterior (occipital) brain activation in alpha frequency might denote the participant’s mentally relaxed state while still maintaining vigilance after dancing (Cantero et al., 2002; Zschocke and Hansen, 2012; Malik and Amin, 2017). This could be beneficial, especially for the creation of an optimal learning state (Manuel et al., 2018), so relaxed wakefulness could be confirmed in connectivity. An increased connectivity creates not only favorable learning conditions, but could also be beneficial in the context of dance therapy, as some neurological diseases are associated with abnormally low connectivity (Du et al., 2018).

### Impact of Imagined Dance With Music

The increased brain activity in temporal (T5) and parietal lobe (Pz, P4) in theta frequency possibly occurs due to the conscious perception and processing of listening to the music (Kolb and Ian, 2015; Malik and Amin, 2017) while visualizing the choreography in space (parietal: spatial cognition) (Kolb and Ian, 2015).

Increased connectivity at beta frequency between central and parietal lobe (C3-P3) might be provoked, because of visualizing the body movements in space. This visualization requires cognitive processing of sensomotoric information (Doppelmayr and Guenter, 2012; Cheron et al., 2016) in central and parietal cortex, which represents i.a. the imagination of body in space, the perception of objects surrounding the body (parietal lobe) or are in direct connection to the execution of limb movements (central lobe) (Kolb and Ian, 2015).

A higher inter-hemispheric connectivity as previously found (Petsche et al., 1993), while solely listening to music or an increased connectivity between T-P (Sarnthein et al., 1997) or FC6-Fp1 (Poikonen et al., 2018b) in music listening laypersons, cannot be confirmed in the present study.

### Impact of Imagined Dance Without Music

The statistically significant decreased brain activity in theta, alpha, beta and gamma frequency, predominantly in frontal (F4), central (Cz) and parietal brain (Pz, P4) lobes, led to the assumption that overall consciousness of participants was reduced. The repetitive memorization of the choreography may have had a soporific or at least a calming effect, particularly reducing brain activity and connectivity in areas of the brain that are normally responsible for conscious processing of memory content (Du Boisgueheneuc et al., 2006; Peng et al., 2018), planning or movement control (prefrontal brain lobe) (Kolb and Ian, 2015). The decreased connectivity in beta frequency between frontopolar-central (Fp1-C3, Fp1-Cz, Fp1-C4, Fp2-Cz), frontal-frontal (F3-F4, F4-F8) and frontal-central (F4-Cz, F8-Cz) brain areas as well as in gamma frequency between frontopolar-parietal (Fp1-Pz) brain lobes, substantiate the assumed interpretations of brain activity. Merely, in alpha frequency occurred an increased connectivity between frontal-temporal electrode pairs (F7-T3), which could be due to the intensive imagination of the choreography that demands the frontal lobe with its feature of persisting in a task and the temporal lobe characteristic of memory associations (Kolb and Ian, 2015).

### Differences Between Test Conditions

Statistically significant differences in brain activity were revealed between the *physically executed dance without music* and the *imagined dance without music* in alpha frequency at frontal (F4) and central (C4) brain lobe as well as in beta frequency at frontal (F4) brain lobe with increased activation in *physically executed dance without music*. Additionally, functional connectivity showed statistically significant differences between these conditions in gamma frequency at frontopolar-frontal electrode pair (Fp1-F4) with increased connectivity in the *physically executed dance without music*. These results, with activation increases solely in the *physically executed dance without music*, led to the assumption that latter demand more planning processes (frontal lobe: planning processes, Kolb and Ian, 2015), like the navigation of the limbs in space or the successive conduction of the choreography, than the pure imagination of that dance. The assumption that the *physically executed dance without music* requires the frontal lobe for planning processes (Kolb and Ian, 2015) can be substantiated in the way that alpha frequency is associated to attentional processes in frontal lobe (Hanslmayr et al., 2011), which is a necessary state for execution of the choreography in the rehearsed manner. The increased connectivity in frontopolar-frontal lobe underpins this interpretation, since gamma frequency is featured to attentional and multisensory processes (Singer and Gray, 1995). Regarding the central brain lobe, the increased alpha activity with its association to attentional processes (Klimesch, 1997) in general, in *physically executed dance without music* could be probably traced back to the executed movements of the choreography (central lobe: movement execution, Kolb and Ian, 2015). The increased frontal beta activity, which is connected to activeness and concentration (Malik and Amin, 2017), substantiates the above mentioned assumption. The decreased brain activity in the *imagined dance without music* could furthermore underpin the assumption of the above mentioned soporific or calming effect of memorizing the dance choreography (Du Boisgueheneuc et al., 2006; Peng et al., 2018).

A clear difference could be observed between *the physically executed* and the *imagined dance without music* in the manner that the *physically executed dance without music* led constantly to brain activity or connectivity increases, while the imagination of the exact same dance led to decreases.

The significant differences in brain activity between the test condition *physically executed dance with* and *without music* were observed in temporal (T4) alpha activity, with increased activation in both conditions. However, alpha activity increased more in *physically executed dance without music*. We assume that participants concentrate more on space and on the navigation of their limbs while executing (temporal lobe: spatial navigation, Kolb and Ian, 2015) the dance without music, since there was no distraction due to music. Probably, the *physically executed dance with music* led to a more automated conduction of the choreography due to music-accompanying movements, which leads to less concentration on space and is therefore reflected in the lower activation of the temporal lobe. Thus, a lower activation in temporal lobe may rather occur while *physically executed dance with music* than while *physically executed dance without music*.

With the analysis of differences between test conditions we could partly confirm the hypothesis of an increased brain activity and functional connectivity in the physically executed dance compared to an imagined dance, since the assumed effects were revealed between the *physically executed* and *imagined dance without music*. Differences between conditions with and without music could be presented between the *physically executed dance with music* and *without music* by a stronger increased activation in brain activity in the *physically executed dance without music*, however, difference was minor.

### Limitations and Future Work

With regard to higher alterations in brain activity and connectivity in the *physically executed dance* compared to the *imagined dance*, it cannot be fully confirmed whether these alterations are caused through metabolic processes or the specific cognitive and perceptive conditions during dance. However, the increased brain activity due to *physically executed dance without music* in more brain lobes and frequency bands as in *physically executed dance with music* do not necessarily trace back to metabolic processes. Nevertheless, the monitoring of metabolic parameters as well as a comparison between different kinds of movement, e.g., dance, running, and cycling, could provide further information about dance-specific effects (e.g., acceleration or rhythm) on the brain.

Furthermore, no objective verification of the participant’s thoughts during the *imagined dance* conditions was conducted. Thus, it can only be assumed that the participants did memorize and imagine the dance choreography and were not in a soporific brain mode, which led to a decreased brain activity and connectivity. An objective validation should be considered in future studies.

With respect to the experimental protocol, there was no EEG rest-measurement prior to the first dance training of the MJD choreography. However, it would be interesting to perform measurements over a longer period of time in order to analyze acute and long-lasting effects of MJD. In addition, recordings were made immediately before and after the interventions in order to find possible underpinnings and explanations for phenomena described in dance therapy studies. Consequently, the investigation of even longer after-effects would be of interest for the sustainability of this type of movement intervention (John and Schollhorn, 2018). Nevertheless, investigating EEG-recordings during the interventions would give further insights of the neurophysiological effects of dance. To conduct recordings during exercise, new EEG-systems with wireless connection are offered, however, they are not free from artifact susceptibility (Bigliassi et al., 2020).

The declaration of this study to a pilot study is based on the small sample size, which occur in several studies (Schneider et al., 2013; Jung et al., 2015; Robertson and Marino, 2015; Edwards et al., 2016). Since we mainly rely on the original Fisher-statistics (Fisher, 1955), extended by the effect sizes according to Neyman-Pearson (Neyman and Pearson, 1933), there is no claim of generalizability (Stegmüller, 1973; Gigerenzer, 2004; Nuzzo, 2014). Rather we show first tendencies of the neurophysiological effects of a physically executed dance in comparison to an imagined dance with or without music. In agreement with Fisher, we conclude, based on the *p* < 0.05 results, that it is worthwhile to pursue this kind of dance research.

Further, the selection of all frequency bands and electrodes resp. electrode pairs could be reduced in future studies on fewer frequencies and electrode positions or electrode combined brain regions to make hypothesis more specific. However, the selection of frequencies and electrodes are not stringent in studies investigating dance, which makes preselection difficult to substantiate.

Apart from the barely researched neurophysiological long-term effects of dance or the comparison between dance and another physical activities the field of group dance is a worthwhile area of research. Most styles are characterized by the fact that they are danced in groups of several people and to our knowledge there are no studies on the difference in the influence of group dance on brain activation compared to solo dance.

## Conclusion

The present pilot study revealed increased brain activity and functional connectivity after a *physically executed dance (with* and *without music)*. Noteworthy differences between a *physically executed* and an *imagined dance* were found, which indicate specific effects of a physically executed dance and may lead to the manifold effects like conscious processes (Malik and Amin, 2017), relaxation (Cantero et al., 2002) and the promotion of executive functions (Bähr and Frotscher, 2014). In addition, the results indicate a specific interaction of movement and music. Music appears to support automated execution of dance movements (or choreographies) that require less brain activity and connectivity. To know more about the diverse and specific effects of different dances on brain and visceral processes would broaden the spectrum of controlled interventions to achieve specific and supportive brain states in combination with other activities (Henz and Schöllhorn, 2017; Henz et al., 2018). Individualized and situationally adapted dances (Burdack et al., 2020) for different diseases, specific patient groups or for the preparation of effective learning and more creative meetings could be applied to optimize the outcome of interventions.

## Data Availability Statement

The raw data supporting the conclusions of this article will be made available by the authors, without undue reservation.

## Ethics Statement

The studies involving human participants were reviewed and approved by Local ethics committee of the Johannes Gutenberg-University of Mainz. The patients/participants provided their written informed consent to participate in this study.

## Author Contributions

JW, FH, NR, AJ, and WS collaborated on preparing the manuscript. JW and WS designed the experiment. JW conducted the data acquisition, data processing and statistical analysis. FH and NR contributed analysis tools. JW, AJ, NR, and WS interpreted the data. JW wrote the manuscript. FH, AJ, NR, and WS critically revised the manuscript. All authors read and approved the final version of the manuscript.

## Conflict of Interest

The authors declare that the research was conducted in the absence of any commercial or financial relationships that could be construed as a potential conflict of interest.
