# Supplementary material for: Electrical Brain Activity and Its Functional Connectivity in the Physical Execution of Modern Jazz Dance
Source: Front Psychol. 2020 Dec 15;11:586076. doi: 10.3389/fpsyg.2020.586076 (PMC7769774; doi:10.3389/fpsyg.2020.586076)
Supplement: Supplementary file 1 [file Table_4.docx]

**Table 4. Significant time effects for electrode pairs of COH.**

|  |  | **Pre-rest^b^** | **Post-rest^b^** | **increased / decreased^c^** | **z^d^** | **p^d^** | **r^d^** |
| --- | --- | --- | --- | --- | --- | --- | --- |
|  |  |  |  |  |  |  |  |
| **da-m^a^** |  |  |  |  |  |  |  |
| Theta | Fp1-F3 | 0.83 | 0.57 | ↑ | -2.05 | .041 | 0.62 |
|  | Fp1-Fz | 0.79 | 0.50 | ↓ | -2.31 | .021 | 0.70 |
|  | Fp2-F3 | 0.54 | 0.34 | ↓ | -1.96 | .050 | 0.59 |
|  | Fp2-Fz | 0.80 | 0.60 | ↓ | -2.31 | .021 | 0.70 |
|  | Fp2-F4 | 0.88 | 0.75 | ↓ | -1.96 | .050 | 0.59 |
|  | F7-T3 | 0.28 | 0.47 | ↑ | -1.96 | .050 | 0.59 |
|  | F8-T6 | 0.14 | 0.06 | ↓ | -2.13 | .033 | 0.64 |
|  | F8-O2 | 0.10 | 0.04 | ↓ | -1.96 | .050 | 0.59 |
|  | C4-P4 | 0.63 | 0.55 | ↓ | -2.05 | .041 | 0.62 |
|  | T4-T6 | 0.56 | 0.48 | ↓ | -1.96 | .050 | 0.59 |
| Alpha | Fp1-F3 | 0.82 | 0.66 | ↓ | -2.22 | .026 | 0.67 |
|  | Fp1-Fz | 0.80 | 0.61 | ↓ | -2.22 | .026 | 0.67 |
|  | C3-C4 | 0.24 | 0.18 | ↓ | -2.05 | .041 | 0.62 |
|  | Cz-P3 | 0.35 | 0.28 | ↓ | -1.96 | .050 | 0.59 |
|  | Cz-P4 | 0.35 | 0.29 | ↓ | -2.22 | .026 | 0.67 |
|  | C4-P3 | 0.23 | 0.17 | ↓ | -2.13 | .033 | 0.64 |
|  | C4-Pz | 0.49 | 0.43 | ↓ | -2.13 | .033 | 0.64 |
|  | C4-P4 | 0.62 | 0.55 | ↓ | -2.22 | .026 | 0.67 |
|  | C4-T6 | 0.83 | 0.25 | ↓ | -1.96 | .050 | 0.59 |
|  | C4-O1 | 1.00 | 0.10 | ↓ | -2.40 | .016 | 0.72 |
|  | C4-O2 | 1.00 | 0.17 | ↓ | -2.40 | .016 | 0.72 |
|  | T4-O1 | 0.16 | 0.12 | ↓ | -2.13 | .033 | 0.64 |
|  | T4-O2 | 0.34 | 0.29 | ↓ | -1.96 | .050 | 0.59 |
|  | T5-P4 | 0.23 | 0.20 | ↓ | -2.05 | .041 | 0.62 |
| Beta | Fp1-F3 | 0.75 | 0.50 | ↓ | -2.05 | .041 | 0.62 |
|  | Fp1-Fz | 0.73 | 0.42 | ↓ | -2.67 | .008 | 0.81 |
|  | Fp2-F3 | 0.41 | 0.25 | ↓ | -1.96 | .050 | 0.59 |
|  | Fp2-F4 | 0.82 | 0.67 | ↓ | -2.49 | .013 | 0.75 |
|  | Fp2-Cz | 0.28 | 0.18 | ↓ | -2.05 | .041 | 0.62 |
|  | F3-C4 | 0.15 | 0.08 | ↓ | -2.22 | .026 | 0.67 |
|  | Fz-O1 | 0.09 | 0.15 | ↑ | -2.40 | .016 | 0.72 |
|  | F8-C3 | 0.09 | 0.06 | ↓ | -2.22 | .026 | 0.67 |
|  | F8-Pz | 0.07 | 0.03 | ↓ | -2.40 | .016 | 0.72 |
|  | C3-Cz | 0.49 | 0.43 | ↓ | -2.13 | .033 | 0.64 |
|  | C3-C4 | 0.19 | 0.12 | ↓ | -2.40 | .016 | 0.72 |
|  | C3-P4 | 0.19 | 0.13 | ↓ | -2.49 | .013 | 0.75 |
|  | Cz-P3 | 0.31 | 0.23 | ↓ | -2.40 | .016 | 0.72 |
|  | Cz-Pz | 0.50 | 0.44 | ↓ | -2.22 | .026 | 0.67 |
|  | Cz-P4 | 0.33 | 0.25 | ↓ | -2.49 | .013 | 0.75 |
|  | C4-T5 | 0.06 | 0.04 | ↓ | -2.13 | .033 | 0.64 |
|  | C4-P3 | 0.19 | 0.12 | ↓ | -2.67 | .008 | 0.81 |
|  | C4-Pz | 0.41 | 0.36 | ↓ | -2.31 | .021 | 0.70 |
|  | C4-P4 | 0.58 | 0.51 | ↓ | -2.85 | .004 | 0.86 |
|  | C4-O1 | 0.10 | 0.05 | ↓ | -2.31 | .021 | 0.70 |
|  | P3-Pz | 0.67 | 0.64 | ↓ | -1.96 | .050 | 0.59 |
|  | P3-P4 | 0.36 | 0.31 | ↓ | -2.22 | .026 | 0.67 |
| Gamma | Fp1-Fz | 0.67 | 0.30 | ↓ | -2.93 | .003 | 0.88 |
|  | Fp1-C3 | 0.32 | 0.14 | ↓ | -2.40 | .016 | 0.72 |
|  | Fp1-Cz | 0.33 | 0.14 | ↓ | -2.49 | .013 | 0.75 |
|  | Fp1-C4 | 0.23 | 0.08 | ↓ | -2.22 | .026 | 0.67 |
|  | Fp1-T5 | 0.16 | 0.07 | ↓ | -1.96 | .050 | 0.59 |
|  | Fp1-Pz | 0.19 | 0.07 | ↓ | -1.96 | .050 | 0.59 |
|  | Fp1-P4 | 0.17 | 0.07 | ↓ | -1.96 | .050 | 0.59 |
|  | Fp2-F3 | 0.43 | 0.18 | ↓ | -2.40 | .016 | 0.72 |
|  | Fp2-F4 | 0.81 | 0.59 | ↓ | -2.22 | .026 | 0.67 |
|  | Fp2-C3 | 0.22 | 0.07 | ↓ | -1.96 | .050 | 0.59 |
|  | Fp2-Cz | 0.33 | 0.14 | ↓ | -2.13 | .033 | 0.64 |
|  | Fp2-C4 | 0.36 | 0.14 | ↓ | -2.22 | .026 | 0.67 |
|  | Fp2-P4 | 0.21 | 0.05 | ↓ | -2.40 | .016 | 0.72 |
|  | Fz-C3 | 0.41 | 0.26 | ↓ | -2.05 | .041 | 0.62 |
|  | Fz-C4 | 0.41 | 0.24 | ↓ | -2.13 | .033 | 0.64 |
|  | Fz-P3 | 0.29 | 0.13 | ↓ | -1.96 | .050 | 0.59 |
|  | Fz-Pz | 0.33 | 0.16 | ↓ | -2.05 | .041 | 0.62 |
|  | Fz-P4 | 0.30 | 0.12 | ↓ | -2.22 | .026 | 0.67 |
|  | Fz-O1 | 0.18 | 0.08 | ↓ | -2.49 | .013 | 0.75 |
|  | Fz-O2 | 0.19 | 0.08 | ↓ | -2.05 | .041 | 0.62 |
|  | F4-C4 | 0.46 | 0.33 | ↓ | -1.96 | .050 | 0.59 |
|  | F4-O1 | 0.17 | 0.06 | ↓ | -2.49 | .013 | 0.75 |
|  | F8-C3 | 0.18 | 0.08 | ↓ | -2.31 | .021 | 0.70 |
|  | F8-Pz | 0.21 | 0.08 | ↓ | -2.40 | .016 | 0.72 |
|  | F8-P4 | 0.25 | 0.08 | ↓ | -2.31 | .021 | 0.70 |
|  | F8-T6 | 0.19 | 0.06 | ↓ | -1.96 | .050 | 0.59 |
|  | Cz-P3 | 0.54 | 0.39 | ↓ | -1.96 | .050 | 0.59 |
|  | Cz-P4 | 0.57 | 0.42 | ↓ | -2.13 | .033 | 0.64 |
|  | C4-P4 | 0.65 | 0.52 | ↓ | -2.22 | .026 | 0.67 |
|  | C4-O1 | 0.22 | 0.08 | ↓ | -2.05 | .041 | 0.62 |
|  |  |  |  |  |  |  |  |
| **da^a^** |  |  |  |  |  |  |  |
| Theta | Fp2-T3 | 0.18 | 0.10 | ↓ | -2.67 | .008 | 0.81 |
|  | Fz-P3 | 0.06 | 0.13 | ↑ | -2.40 | .016 | 0.72 |
|  | F8-C3 | 0.17 | 0.10 | ↓ | -1.96 | .050 | 0.59 |
| Alpha |  |  |  |  |  |  |  |
|  | Fp1-O1 | 0.27 | 0.37 | ↑ | -2.13 | .033 | 0.64 |
|  | Fp2-P3 | 0.14 | 0.18 | ↑ | -2.40 | .016 | 0.72 |
|  | Fp2-O1 | 0.28 | 0.38 | ↑ | -2.40 | .016 | 0.72 |
|  | Fp2-O2 | 0.23 | 0.31 | ↑ | -2.31 | .021 | 0.70 |
|  | Fz-P3 | 0.10 | 0.16 | ↑ | -1.96 | .050 | 0.59 |
|  | Fz-P4 | 0.10 | 0.16 | ↑ | -1.96 | .050 | 0.59 |
|  | Fz-T6 | 0.17 | 0.24 | ↑ | -1.96 | .050 | 0.59 |
|  | Fz-O1 | 0.16 | 0.31 | ↑ | -2.13 | .033 | 0.64 |
|  | Fz-O2 | 0.17 | 0.28 | ↑ | -2.22 | .026 | 0.67 |
|  | F4-P3 | 0.10 | 0.16 | ↑ | -2.05 | .041 | 0.62 |
|  | F4-O1 | 0.17 | 0.29 | ↑ | -2.22 | .026 | 0.67 |
|  | F4-O2 | 0.14 | 0.23 | ↑ | -2.22 | .026 | 0.67 |
|  | C3-T6 | 0.07 | 0.10 | ↑ | -2.22 | .026 | 0.67 |
|  | P3-Pz | 0.69 | 0.66 | ↓ | -1.96 | .050 | 0.59 |
|  | O1-O2 | 0.65 | 0.69 | ↑ | -2.13 | .033 | 0.64 |
| Beta | F7-F3 | 0.45 | 0.58 | ↑ | -2.05 | .041 | 0.62 |
|  | F8-C3 | 0.12 | 0.09 | ↓ | -2.67 | .008 | 0.81 |
|  | O1-O2 | 0.50 | 0.61 | ↑ | -2.40 | .016 | 0.72 |
| Gamma | Fp1-O2 | 0.20 | 0.12 | ↓ | -2.40 | .016 | 0.72 |
|  | F7-F3 | 0.41 | 0.59 | ↑ | -2.22 | .026 | 0.67 |
|  | Fz-F4 | 0.61 | 0.53 | ↓ | -2.05 | .041 | 0.62 |
|  |  |  |  |  |  |  |  |
| **im-m^a^** |  |  |  |  |  |  |  |
| Theta | F7-F8 | 0.11 | 0.29 | ↑ | -2.13 | .033 | 0.64 |
|  | F7-T3 | 0.38 | 0.47 | ↑ | -1.96 | .050 | 0.59 |
|  | F7-T4 | 0.12 | 0.24 | ↑ | -2.58 | .010 | 0.78 |
| Alpha | F7-T3 | 0.39 | 0.47 | ↑ | -1.96 | .050 | 0.59 |
|  | F4-F8 | 0.65 | 0.54 | ↓ | -2.05 | .041 | 0.62 |
| Beta | Fp1-F8 | 0.19 | 0.12 | ↓ | -2.13 | .033 | 0.64 |
|  | F7-F8 | 0.07 | 0.23 | ↑ | -2.31 | .021 | 0.70 |
|  | F4-F8 | 0.57 | 0.42 | ↓ | -2.22 | .026 | 0.67 |
|  | C3-P3 | 0.54 | 0.58 | ↑ | -2.05 | .041 | 0.62 |
|  | C4-O2 | 0.16 | 0.21 | ↑ | -1.96 | .050 | 0.59 |
|  | T5-P3 | 0.64 | 0.69 | ↑ | -2.40 | .016 | 0.72 |
|  | T5-Pz | 0.29 | 0.33 | ↑ | -2.05 | .041 | 0.62 |
|  | P3-Pz | 0.67 | 0.70 | ↑ | -2.13 | .033 | 0.64 |
|  | P3-P4 | 0.38 | 0.41 | ↑ | -1.96 | .050 | 0.59 |
|  | Pz-O1 | 0.38 | 0.47 | ↑ | -2.49 | .013 | 0.75 |
|  | T6-O2 | 0.67 | 0.73 | ↑ | -1.96 | .050 | 0.59 |
| Gamma | F4-F8 | 0.56 | 0.36 | ↓ | -2.13 | .033 | 0.64 |
|  | F8-C3 | 0.17 | 0.12 | ↓ | -2.13 | .033 | 0.64 |
|  | F8-P3 | 0.12 | 0.07 | ↓ | -2.22 | .026 | 0.67 |
|  | F8-Pz | 0.14 | 0.08 | ↓ | -2.40 | .016 | 0.72 |
|  | F8-P4 | 0.16 | 0.09 | ↓ | -2.13 | .033 | 0.64 |
|  | F8-O2 | 0.19 | 0.05 | ↓ | -2.05 | .041 | 0.62 |
|  | C3-P3 | 0.58 | 0.66 | ↑ | -1.96 | .050 | 0.59 |
|  | C4-T6 | 0.28 | 0.38 | ↑ | -2.13 | .033 | 0.64 |
|  | P3-Pz | 0.70 | 0.75 | ↑ | -2.05 | .041 | 0.62 |
|  | P4-O2 | 0.43 | 0.58 | ↑ | -2.22 | .026 | 0.67 |
|  | T6-O2 | 0.58 | 0.72 | ↑ | -1.96 | .050 | 0.59 |
|  |  |  |  |  |  |  |  |
| **im^a^** |  |  |  |  |  |  |  |
| Theta | Fp1-F4 | 0.63 | 0.52 | ↓ | -2.05 | .041 | 0.62 |
|  | Fp1-F8 | 0.38 | 0.18 | ↓ | -2.13 | .033 | 0.64 |
|  | Fp1-C3 | 0.37 | 0.24 | ↓ | -2.67 | .008 | 0.81 |
|  | Fp1-Cz | 0.44 | 0.30 | ↓ | -2.67 | .008 | 0.81 |
|  | Fp2-F3 | 0.55 | 0.44 | ↓ | -2.13 | .033 | 0.64 |
|  | Fp2-Fz | 0.75 | 0.64 | ↓ | -2.22 | .026 | 0.67 |
|  | F7-C4 | 0.14 | 0.08 | ↓ | -2.31 | .021 | 0.70 |
|  | F3-F4 | 0.61 | 0.49 | ↓ | -1.96 | .050 | 0.59 |
|  | F3-C3 | 0.54 | 0.44 | ↓ | -2.22 | .026 | 0.67 |
|  | Fz-Cz | 0.62 | 0.52 | ↓ | -2.31 | .021 | 0.70 |
|  | Fz-C4 | 0.41 | 0.30 | ↓ | -1.96 | .050 | 0.59 |
|  | F4-Cz | 0.54 | 0.44 | ↓ | -1.96 | .050 | 0.59 |
|  | F8-Cz | 0.33 | 0.18 | ↓ | -1.96 | .050 | 0.59 |
| Alpha | Fp1-F4 | 0.61 | 0.50 | ↓ | -2.31 | .021 | 0.70 |
|  | Fp1-C3 | 0.30 | 0.24 | ↓ | -2.05 | .041 | 0.62 |
|  | Fp1-Cz | 0.40 | 0.30 | ↓ | -2.40 | .016 | 0.72 |
|  | Fp2-Fz | 0.74 | 0.66 | ↓ | -2.31 | .021 | 0.70 |
|  | F7-T3 | 0.37 | 0.43 | ↓ | -2.05 | .041 | 0.62 |
|  | F7-Cz | 0.28 | 0.21 | ↓ | -2.13 | .033 | 0.64 |
|  | Fz-Cz | 0.62 | 0.55 | ↓ | -2.13 | .033 | 0.64 |
|  | F8-T6 | 0.13 | 0.17 | ↑ | -1.96 | .050 | 0.59 |
|  | T3-C4 | 0.14 | 0.09 | ↓ | -2.93 | .003 | 0.88 |
| Beta | Fp1-F4 | 0.56 | 0.38 | ↓ | -2.13 | .033 | 0.64 |
|  | Fp1-F8 | 0.30 | 0.12 | ↓ | -2.13 | .033 | 0.64 |
|  | Fp1-C3 | 0.30 | 0.19 | ↓ | -2.93 | .003 | 0.88 |
|  | Fp1-Cz | 0.39 | 0.25 | ↓ | -2.58 | .010 | 0.78 |
|  | Fp1-C4 | 0.20 | 0.10 | ↓ | -2.67 | .008 | 0.81 |
|  | Fp2-F3 | 0.43 | 0.32 | ↓ | -2.05 | .041 | 0.62 |
|  | Fp2-Fz | 0.67 | 0.54 | ↓ | -2.40 | .016 | 0.72 |
|  | Fp2-Cz | 0.32 | 0.23 | ↓ | -2.13 | .033 | 0.64 |
|  | F3-F4 | 0.50 | 0.34 | ↓ | -2.40 | .016 | 0.72 |
|  | Fz-F4 | 0.78 | 0.69 | ↓ | -2.13 | .033 | 0.64 |
|  | Fz-F8 | 0.36 | 0.19 | ↓ | -2.13 | .033 | 0.64 |
|  | Fz-Cz | 0.58 | 0.51 | ↓ | -2.31 | .021 | 0.70 |
|  | Fz-C4 | 0.32 | 0.25 | ↓ | -2.05 | .041 | 0.62 |
|  | F4-F8 | 0.60 | 0.45 | ↓ | -2.05 | .041 | 0.62 |
|  | F4-Cz | 0.46 | 0.37 | ↓ | -1.96 | .050 | 0.59 |
|  | F8-Cz | 0.27 | 0.12 | ↓ | -2.22 | .026 | 0.67 |
|  | T3-C4 | 0.10 | 0.06 | ↓ | -1.96 | .050 | 0.59 |
|  | O1-O2 | 0.58 | 0.65 | ↑ | -2.13 | .033 | 0.64 |
| Gamma | Fp1-C3 | 0.39 | 0.21 | ↓ | -2.40 | .016 | 0.72 |
|  | Fp1-Cz | 0.41 | 0.25 | ↓ | -2.22 | .026 | 0.67 |
|  | Fp1-C4 | 0.31 | 0.16 | ↓ | -2.13 | .033 | 0.64 |
|  | Fp1-P3 | 0.22 | 0.10 | ↓ | -1.96 | .050 | 0.59 |
|  | Fp1-Pz | 0.24 | 0.11 | ↓ | -2.40 | .016 | 0.72 |
|  | Fp2-Fz | 0.63 | 0.50 | ↓ | -2.13 | .033 | 0.64 |
|  | Fp2-C3 | 0.28 | 0.14 | ↓ | -2.31 | .021 | 0.70 |
|  | Fp2-Cz | 0.37 | 0.24 | ↓ | -2.31 | .021 | 0.70 |
|  | Fp2-T4 | 0.30 | 0.16 | ↓ | -2.22 | .026 | 0.67 |
|  | F3-F4 | 0.51 | 0.32 | ↓ | -2.05 | .041 | 0.62 |
|  | Fz-C3 | 0.50 | 0.37 | ↓ | -2.22 | .026 | 0.67 |
|  | Fz-Cz | 0.64 | 0.55 | ↓ | -2.13 | .033 | 0.64 |
|  | Fz-P3 | 0.34 | 0.24 | ↓ | -2.13 | .033 | 0.64 |
|  | Fz-Pz | 0.40 | 0.28 | ↓ | -2.22 | .026 | 0.67 |
|  | F8-T3 | 0.13 | 0.24 | ↑ | -1.96 | .050 | 0.59 |
|  | F8-Cz | 0.28 | 0.15 | ↓ | -2.05 | .041 | 0.62 |
|  | Cz-Pz | 0.71 | 0.64 | ↓ | -2.13 | .033 | 0.64 |
|  | P4-T6 | 0.68 | 0.59 | ↓ | -2.05 | .041 | 0.62 |
|  | P4-O2 | 0.63 | 0.55 | ↓ | -1.96 | .050 | 0.59 |

**Note**: Statistically significant time effects of the electrode pairs of COH with presentation of the COH values.

^a^da-m: physically-executed dance with music, da: physically-executed dance without music, im-m: imagined dance with music, im: imagined dance without music

^a^pre- and post-rest values of COH

^c^arrow up: connectivity increased from pre- to post-rest measurement, arrow down: connectivity decreased from pre- to post-rest measurement

^d^z-value of the Wilcoxon-test, p-value, r-value of the effect size
