# Supplementary material for: Electrical Brain Activity and Its Functional Connectivity in the Physical Execution of Modern Jazz Dance
Source: Front Psychol. 2020 Dec 15;11:586076. doi: 10.3389/fpsyg.2020.586076 (PMC7769774; doi:10.3389/fpsyg.2020.586076)
Supplement: Supplementary file 2 [file Table_5.docx]

**Table 5. Significant time effects for electrode pairs of ICOH.**

|  |  | **Pre-rest^b^** | **Post-rest^b^** | **increased / decreased^c^** | **z^d^** | **p^d^** | **r^d^** |
| --- | --- | --- | --- | --- | --- | --- | --- |
|  |  |  |  |  |  |  |  |
| **da-m^a^** |  |  |  |  |  |  |  |
| Theta | Fp2-F7 | 0.04 | 0.03 | ↓ | -2.49 | .013 | 0.75 |
|  | F7-F3 | 0.05 | 0.04 | ↓ | -1.96 | .050 | 0.59 |
|  | F7-T3 | 0.04 | 0.02 | ↓ | -2.05 | .041 | 0.62 |
|  | F8-C4 | 0.04 | 0.02 | ↓ | -2.13 | .033 | 0.64 |
|  | C3-T4 | 0.03 | 0.05 | ↑ | -1.96 | .050 | 0.59 |
|  | C3-P4 | 0.02 | 0.04 | ↑ | -2.05 | .041 | 0.62 |
|  | Cz-P4 | 0.02 | 0.04 | ↑ | -2.05 | .041 | 0.62 |
|  | T4-P3 | 0.03 | 0.05 | ↑ | -2.13 | .033 | 0.64 |
| Alpha | Fp2-F4 | 0.04 | 0.03 | ↓ | -2.13 | .033 | 0.64 |
|  | Fp2-F8 | 0.07 | 0.04 | ↓ | -2.40 | .016 | 0.72 |
|  | F3-F4 | 0.06 | 0.04 | ↓ | -2.22 | .026 | 0.67 |
|  | F3-C3 | 0.06 | 0.07 | ↑ | -1.96 | .050 | 0.59 |
| Beta | F7-T5 | 0.02 | 0.04 | ↑ | -2.31 | .021 | 0.70 |
|  | F8-P3 | 0.03 | 0.02 | ↓ | -2.05 | .041 | 0.62 |
|  | T4-Pz | 0.02 | 0.03 | ↑ | -2.31 | .021 | 0.70 |
| Gamma | F7-C3 | 0.02 | 0.01 | ↓ | -1.96 | .050 | 0.59 |
|  | F7-Pz | 0.04 | 0.02 | ↓ | -2.05 | .041 | 0.62 |
|  | T6-O2 | 0.01 | 0.03 | ↑ | -2.13 | .033 | 0.64 |
|  |  |  |  |  |  |  |  |
| **da^a^** |  |  |  |  |  |  |  |
| Theta | F7-F3 | 0.05 | 0.02 | ↓ | -2.40 | .016 | 0.72 |
|  | F7-Fz | 0.06 | 0.03 | ↓ | -2.49 | .013 | 0.75 |
| Alpha | Fp1-T5 | 0.14 | 0.11 | ↓ | -2.05 | .041 | 0.62 |
|  | Fp1-O1 | 0.12 | 0.10 | ↓ | -2.31 | .021 | 0.70 |
|  | T3-Cz | 0.09 | 0.06 | ↓ | -2.31 | .021 | 0.70 |
|  | T3-C4 | 0.07 | 0.04 | ↓ | -2.40 | .016 | 0.72 |
|  | T3-T4 | 0.06 | 0.03 | ↓ | -2.22 | .026 | 0.67 |
|  | T3-P4 | 0.09 | 0.06 | ↓ | -2.67 | .008 | 0.81 |
|  | T3-T6 | 0.08 | 0.05 | ↓ | -2.40 | .016 | 0.72 |
|  | C3-C4 | 0.06 | 0.03 | ↓ | -2.29 | .022 | 0.69 |
|  | P3-Pz | 0.06 | 0.03 | ↓ | -1.96 | .050 | 0.59 |
| Beta | Fp2-T5 | 0.03 | 0.04 | ↑ | -2.22 | .026 | 0.67 |
|  | F7-C4 | 0.03 | 0.02 | ↓ | -2.22 | .026 | 0.67 |
|  | F3-C4 | 0.03 | 0.02 | ↓ | -1.96 | .050 | 0.59 |
|  | F8-P4 | 0.02 | 0.04 | ↑ | -2.05 | .041 | 0.62 |
|  | C3-C4 | 0.03 | 0.01 | ↓ | -2.05 | .041 | 0.62 |
|  | C3-P4 | 0.03 | 0.02 | ↓ | -1.96 | .050 | 0.59 |
|  | C3-T6 | 0.03 | 0.02 | ↓ | -1.96 | .050 | 0.59 |
|  | P3-P4 | 0.02 | 0.01 | ↓ | -2.13 | .033 | 0.64 |
| Gamma | Fp1-Fp2 | 0.02 | 0.01 | ↓ | -2.13 | .033 | 0.64 |
|  | Fp1-F4 | 0.03 | 0.01 | ↓ | -2.31 | .021 | 0.70 |
|  | Fp1-Pz | 0.02 | 0.01 | ↓ | -2.58 | .010 | 0.78 |
|  | F3-F8 | 0.02 | 0.01 | ↓ | -2.31 | .021 | 0.70 |
|  | F8-Cz | 0.02 | 0.01 | ↓ | -2.49 | .013 | 0.75 |
|  |  |  |  |  |  |  |  |
| **im-m^a^** |  |  |  |  |  |  |  |
| Theta | Fp2-Fz | 0.03 | 0.01 | ↓ | -2.22 | .026 | 0.67 |
|  | F8-Pz | 0.02 | 0.04 | ↑ | -2.31 | .021 | 0.70 |
|  | P3-Pz | 0.01 | 0.03 | ↑ | -2.40 | .016 | 0.72 |
| Alpha | T3-P4 | 0.07 | 0.11 | ↑ | -2.76 | .006 | 0.83 |
|  | C3-C4 | 0.04 | 0.07 | ↑ | -2.22 | .026 | 0.67 |
|  | T4-T6 | 0.11 | 0.08 | ↓ | -2.49 | .013 | 0.75 |
| Beta | Fp1-O1 | 0.03 | 0.04 | ↑ | -2.49 | .013 | 0.75 |
|  | Fp2-T4 | 0.03 | 0.04 | ↑ | -1.96 | .050 | 0.59 |
|  | F3-T4 | 0.02 | 0.03 | ↑ | -2.13 | .033 | 0.64 |
|  | Fz-C4 | 0.02 | 0.03 | ↑ | -2.05 | .041 | 0.62 |
|  | Fz-T5 | 0.05 | 0.06 | ↑ | -2.13 | .033 | 0.64 |
|  | Fz-O1 | 0.03 | 0.04 | ↑ | -2.22 | .026 | 0.67 |
|  | C3-P3 | 0.03 | 0.02 | ↓ | -2.05 | .041 | 0.62 |
|  | C4-P3 | 0.04 | 0.03 | ↓ | -2.13 | .033 | 0.64 |
|  | T4-P3 | 0.03 | 0.02 | ↓ | -2.05 | .041 | 0.62 |
|  | T5-T6 | 0.03 | 0.01 | ↓ | -2.67 | .008 | 0.81 |
|  | O1-O2 | 0.01 | 0.01 | = | -2.13 | .033 | 0.64 |
| Gamma | Fz-F4 | 0.01 | 0.01 | = | -2.05 | .041 | 0.62 |
|  | F4-C4 | 0.02 | 0.01 | ↓ | -2.40 | .016 | 0.72 |
|  | F4-T6 | 0.02 | 0.02 | = | -2.05 | .041 | 0.62 |
|  | F4-O1 | 0.01 | 0.02 | ↑ | -2.58 | .010 | 0.78 |
|  | F8-T5 | 0.02 | 0.02 | = | -2.40 | .016 | 0.72 |
|  | F8-O1 | 0.02 | 0.03 | ↑ | -2.76 | .006 | 0.83 |
|  | T3-Pz | 0.01 | 0.03 | ↑ | -1.96 | .050 | 0.59 |
|  | P3-O2 | 0.01 | 0.02 | ↑ | -2.49 | .013 | 0.75 |
|  |  |  |  |  |  |  |  |
| **im^a^** |  |  |  |  |  |  |  |
| Theta | Fp1-F7 | 0.02 | 0.03 | ↑ | -2.49 | .013 | 0.75 |
|  | F7-O2 | 0.05 | 0.03 | ↓ | -2.40 | .016 | 0.72 |
|  | C3-P4 | 0.03 | 0.02 | ↓ | -2.49 | .013 | 0.75 |
|  | P3-Pz | 0.01 | 0.03 | ↑ | -2.31 | .021 | 0.70 |
| Alpha | F7-T3 | 0.05 | 0.08 | ↑ | -2.49 | .013 | 0.75 |
|  | F4-T3 | 0.08 | 0.11 | ↑ | -2.31 | .021 | 0.70 |
|  | T3-C4 | 0.05 | 0.08 | ↑ | -1.96 | .050 | 0.59 |
|  | T3-Pz | 0.05 | 0.09 | ↑ | -2.22 | .026 | 0.67 |
|  | P3-Pz | 0.04 | 0.06 | ↑ | -1.96 | .050 | 0.59 |
| Beta | Fp1-C3 | 0.03 | 0.04 | ↑ | -1.96 | .050 | 0.59 |
|  | Fp1-Cz | 0.01 | 0.02 | ↑ | -1.96 | .050 | 0.59 |
|  | Fp1-C4 | 0.02 | 0.03 | ↑ | -2.13 | .033 | 0.64 |
|  | Fp1-Pz | 0.02 | 0.03 | ↑ | -1.96 | .050 | 0.59 |
|  | Fp2-Cz | 0.01 | 0.03 | ↑ | -2.49 | .013 | 0.75 |
|  | F7-F8 | 0.01 | 0.02 | ↑ | -1.96 | .050 | 0.59 |
|  | F3-F4 | 0.01 | 0.02 | ↑ | -2.05 | .041 | 0.62 |
|  | Fz-Cz | 0.01 | 0.02 | ↑ | -2.58 | .010 | 0.78 |
| Beta | Fz-Pz | 0.02 | 0.04 | ↑ | -2.93 | .003 | 0.88 |
|  | F4-F8 | 0.01 | 0.02 | ↑ | -2.05 | .041 | 0.62 |
|  | F4-Cz | 0.01 | 0.03 | ↑ | -2.40 | .016 | 0.72 |
|  | F8-T3 | 0.04 | 0.02 | ↓ | -2.49 | .013 | 0.75 |
|  | F8-Cz | 0.01 | 0.02 | ↑ | -1.96 | .050 | 0.59 |
|  | C3-P4 | 0.02 | 0.03 | ↑ | -2.13 | .033 | 0.64 |
|  | Cz-T4 | 0.03 | 0.04 | ↑ | -2.22 | .026 | 0.67 |
|  | Cz-Pz | 0.02 | 0.03 | ↑ | -2.31 | .021 | 0.70 |
|  | P3-T6 | 0.02 | 0.03 | ↑ | -2.67 | .008 | 0.81 |
| Gamma | Fp1-F4 | 0.01 | 0.02 | ↑ | -1.96 | .050 | 0.59 |
|  | Fp1-Pz | 0.03 | 0.02 | ↓ | -2.05 | .041 | 0.62 |
|  | Fp2-Pz | 0.03 | 0.01 | ↓ | -2.13 | .033 | 0.64 |
|  | F8-P3 | 0.03 | 0.01 | ↓ | -2.49 | .013 | 0.75 |
|  | F8-Pz | 0.03 | 0.01 | ↓ | -2.05 | .041 | 0.62 |
|  | Cz-Pz | 0.02 | 0.02 | = | -2.13 | .033 | 0.64 |
|  | T4-T5 | 0.02 | 0.01 | ↓ | -2.05 | .041 | 0.62 |
|  | P3-P4 | 0.02 | 0.01 | ↓ | -2.40 | .016 | 0.72 |

**Note**: Statistically significant time effects of the electrode pairs of ICOH with presentation of the ICOH values.

^a^ da-m: physically-executed dance with music, da: physically-executed dance without music, im-m: imagined dance with music, im: imagined dance without music

^b^pre- and post-rest values of ICOH

^c^z-value of the Wilcoxon-test, p-value, r-value of the effect size
