# Supplementary material for: Electrical Brain Activity and Its Functional Connectivity in the Physical Execution of Modern Jazz Dance
Source: Front Psychol. 2020 Dec 15;11:586076. doi: 10.3389/fpsyg.2020.586076 (PMC7769774; doi:10.3389/fpsyg.2020.586076)
Supplement: Supplementary file 3 [file Table_6.docx]

**Table 6. Significant differences between test conditions of COH.**

|  |  |  |  |  |  | **Post-hoc comparisons** | | |
| --- | --- | --- | --- | --- | --- | --- | --- | --- |
|  |  | **χ2 (df)^b^** | **global p^b^** | **Diff-rest 1^c^** | **Diff-rest 2^c^** | **p^b^** | **z^b^** | **r^b^** |
| **da-m*pa^a^** |  |  |  |  |  |  |  |  |
| Alpha | F7-T3 | 9.66 (3) | .022 | 0.102 | -0.065 | .049 | 1.46 | 0.44 |
| Gamma | Cz-P4 | 11.18 (3) | .011 | -0.143 | 0.041 | .018 | -1.64 | 0.49 |
|  | P3-P4 | 9.33 (3) | .025 | -0.139 | 0.011 | .049 | -1.46 | 0.44 |
|  |  |  |  |  |  |  |  |  |
| **da-m*im-m^a^** |  |  |  |  |  |  |  |  |
| Alpha | Fp1-F7 | 7.91 (3) | .048 | 0.089 | -0.099 | .049 | 1.46 | 0.44 |
| Gamma | Cz-P4 | 11.18 (3) | .011 | -0.143 | 0.043 | .049 | -1.46 | 0.44 |
|  | P3-P4 | 9.33 (3) | .025 | -0.139 | 0.068 | .049 | -1.46 | 0.44 |
| Beta | F4-F8 | 11.18 (3) | .011 | 0.082 | -0.150 | .018 | 1.64 | 0.49 |
|  | Cz-P3 | 8.78 (3) | .032 | -0.082 | 0.054 | .030 | -1.55 | 0.47 |
|  | Cz-O1 | 10.31 (3) | .016 | -0.032 | 0.045 | .010 | -1.73 | 0.52 |
|  | Cz-O2 | 8.13 (3) | .043 | -0.017 | 0.026 | .030 | -1.55 | 0.47 |
|  | C4-P3 | 8.13 (3) | .043 | -0.069 | 0.055 | .030 | -1.55 | 0.47 |
|  | C4-P4 | 10.31 (3) | .016 | -0.065 | 0.029 | .010 | -1.73 | 0.52 |
|  | P3-P4 | 9.33 (3) | .025 | -0.049 | 0.032 | .018 | -1.64 | 0.49 |
|  | Pz-O1 | 12.27 (3) | .007 | -0.038 | 0.092 | .010 | -1.73 | 0.52 |
|  | P4-O1 | 9.00 (3) | .029 | -0.044 | 0.054 | .030 | -1.55 | 0.47 |
| Gamma | Fz-T5 | 11.40 (3) | .010 | -0.057 | 0.067 | .030 | -1.55 | 0.47 |
|  | Fz-P3 | 9.00 (3) | .029 | -0.155 | 0.091 | .030 | -1.55 | 0.47 |
|  | F4-P3 | 8.46 (3) | .037 | -0.121 | 0.094 | .049 | -1.46 | 0.44 |
|  | C3-P3 | 9.22 (3) | .027 | -0.121 | -0.001 | .049 | -1.46 | 0.44 |
|  | C3-O1 | 9.22 (3) | .027 | -0.121 | 0.114 | .049 | -1.46 | 0.44 |
|  | Cz-P3 | 13.36 (3) | .004 | -0.149 | 0.102 | .003 | -1.91 | 0.58 |
|  | Cz-O1 | 8.78 (3) | .032 | -0.152 | 0.110 | .030 | -1.55 | 0.47 |
|  | C4-P3 | 7.91 (3) | .048 | -0.155 | 0.103 | .049 | -1.46 | 0.44 |
|  | P3-Pz | 8.13 (3) | .043 | -0.084 | 0.053 | .030 | -1.55 | 0.47 |
|  |  |  |  |  |  |  |  |  |
| **da-m*im^a^** |  |  |  |  |  |  |  |  |
| Beta | F4-F8 | 11.18 (3) | .011 | 0.082 | -0.153 | .030 | 1.55 | 0.47 |
|  | Fp1-F8 | 8.78 (3) | .032 | 0.069 | -0.175 | .049 | 1.46 | 0.44 |
|  | O1-O2 | 10.11 (3) | .018 | -0.051 | 0.079 | .010 | -1.73 | 0.52 |
|  |  |  |  |  |  |  |  |  |
| **da*im-m^a^** |  |  |  |  |  |  |  |  |
| Alpha | F4-O1 | 8.13 (3) | .043 | 0.123 | -0.029 | .030 | 1.55 | 0.47 |
| Gamma | Fz-T5 | 11.40 (3) | .010 | -0.065 | 0.067 | .030 | -1.55 | 0.47 |
|  | T6-O2 | 8.78 (3) | .032 | -0.160 | 0.145 | .030 | -1.55 | 0.47 |
|  |  |  |  |  |  |  |  |  |
| **da*im^a^** |  |  |  |  |  |  |  |  |
| Beta | Fz-C4 | 10.31 (3) | .016 | -0.008 | -0.072 | .030 | 1.55 | 0.47 |
| Gamma | Fp1-F4 | 9.00 (3) | .029 | 0.134 | -0.163 | .018 | 1.64 | 0.49 |
|  |  |  |  |  |  |  |  |  |
| **im-m*im^a^** |  |  |  |  |  |  |  |  |
| Beta | C4-T5 | 8.78 (3) | .032 | 0.028 | -0.031 | .049 | 1.46 | 0.44 |
|  | Pz-O1 | 12.27 (3) | .007 | -0.038 | 0.092 | .049 | 1.46 | 0.44 |

**Note**: Statistically significant differences between test conditions with presentation of the Diff-rest values of COH.

Left column descriptive values, right column – statistical values.

^a^statistical differences between test condition pairs, da-m: physically-executed dance with music, da: physically-executed dance without music, im-m: imagined dance with music, im: imagined dance without music

^b^ χ2 with with degrees of freedom of Friedman-test, r-value effect size of the Friedman-test, z-value of the Friedman-test.

^c^Diff-rest 1: difference value from pre- to post-rest-measurement of the left test condition from test condition pair, Diff-rest 2: difference value from pre- to post-rest-measurement of the right test condition from test condition pair.
