# Supplementary material for: Electrical Brain Activity and Its Functional Connectivity in the Physical Execution of Modern Jazz Dance
Source: Front Psychol. 2020 Dec 15;11:586076. doi: 10.3389/fpsyg.2020.586076 (PMC7769774; doi:10.3389/fpsyg.2020.586076)
Supplement: Supplementary file 4 [file Table_7.docx]

**Table 7. Significant differences between test conditions of ICOH.**

|  | | | |  |  | **Post-hoc comparisons** | | |
| --- | --- | --- | --- | --- | --- | --- | --- | --- |
|  |  | **χ2 (df)^b^** | **global p^b^** | **Diff-rest 1^c^** | **Diff-rest 2^c^** | **p^b^** | **z^b^** | **r^b^** |
| **da-m*pa^a^** |  |  |  |  |  |  |  |  |
| Theta | P3-T6 | 10.90 (3) | .018 | 0.014 | -0.022 | .010 | 1.73 | 0.52 |
| Beta | F7-T5 | 9.76 (3) | .021 | 0.021 | 0.004 | .049 | 1.46 | 0.44 |
| Gamma | Fp1-T5 | 9.44 (3) | .024 | 0.005 | -0.014 | .030 | 1.55 | 0.47 |
|  | F8-Cz | 8.23 (3) | .043 | 0.002 | -0.013 | .030 | 1.55 | 0.47 |
|  | C3-T6 | 9.00 (3) | .029 | -0.012 | 0.010 | .018 | -1.64 | 0.49 |
|  |  |  |  |  |  |  |  |  |
| **da-m*im-m^a^** |  |  |  |  |  |  |  |  |
| Alpha | F3-F4 | 9.00 (3) | .029 | -0.018 | 0.016 | .018 | -1.64 | 0.49 |
| Beta | F7-T5 | 9.76 (3) | .021 | 0.021 | 0.002 | .049 | 1.46 | 0.44 |
|  |  |  |  |  |  |  |  |  |
| **da-m*im^a^** |  |  |  |  |  |  |  |  |
| Beta | C3-P3 | 9.87 (3) | .020 | 0.011 | -0.008 | .010 | 1.73 | 0.52 |
|  | C3-P4 | 10.96 (3) | .012 | -0.009 | 0.002 | .030 | -1.55 | 0.47 |
|  | C4-O1 | 9.87 (3) | .020 | 0.004 | 0.000 | .049 | 1.46 | 0.44 |
| Gamma | P3-P4 | 9.76 (3) | .021 | 0.010 | -0.013 | .018 | -1.64 | 0.49 |
|  |  |  |  |  |  |  |  |  |
| **da*im-m^a^** |  |  |  |  |  |  |  |  |
| Theta | P3-O2 | 8.78 (3) | .032 | -0.012 | 0.014 | .049 | -1.46 | 0.44 |
| Alpha | T3-C4 | 11.18 (3) | .011 | -0.040 | 0.018 | .030 | -1.55 | 0.47 |
|  | T3-Pz | 10.96 (3) | .012 | -0.034 | 0.023 | .049 | -1.46 | 0.44 |
|  | T3-P4 | 8.02 (3) | .046 | -0.035 | 0.033 | .049 | -1.46 | 0.44 |
|  | C3-C4 | 14.67 (3) | .002 | -0.029 | 0.026 | .002 | -2.00 | 0.60 |
| Gamma | Fp1-Pz | 8.78 (3) | .032 | -0.013 | 0.012 | .049 | -1.46 | 0.44 |
|  |  |  |  |  |  |  |  |  |
| **da*im^a^** |  |  |  |  |  |  |  |  |
| Alpha | T3-Cz | 8.78 (3) | .032 | -0.039 | 0.043 | .049 | -1.46 | 0.44 |
|  | P3-Pz | 10.31 (3) | .016 | -0.024 | 0.011 | .018 | -1.64 | 0.49 |
| Beta | Fp1-Fp2 | 7.91 (3) | .048 | -0.007 | 0.005 | .049 | -1.46 | 0.44 |
|  | Fp2-Cz | 8.35 (3) | .039 | 0.001 | 0.020 | .030 | -1.55 | 0.47 |
|  | C4-O1 | 9.87 (3) | .020 | 0.004 | 0.000 | .018 | -1.64 | 0.49 |
| Gamma | Fp1-F4 | 9.87 (3) | .020 | -0.014 | 0.012 | .030 | -1.55 | 0.47 |
|  |  |  |  |  |  |  |  |  |
| **im-m*im^a^** |  |  |  |  |  |  |  |  |
| Theta | T5-T6 | 10.31 (3) | .016 | 0.016 | -0.011 | .030 | 1.55 | 0.47 |
| Alpha | C3-C4 | 14.67 (3) | .002 | 0.026 | -0.026 | .030 | 1.55 | 0.47 |
| Gamma | F8-T5 | 7.91 (3) | .048 | 0.008 | -0.009 | .030 | 1.55 | 0.47 |
|  | F8-P3 | 12.06 (3) | .007 | 0.007 | -0.023 | .003 | 1.91 | 0.58 |

**Note**: Statistically significant differences between test conditions with presentation of the Diff-rest values of ICOH.

Left column descriptive values, right column – statistical values.

^a^statistical differences between test condition pairs, da-m: physically-executed dance with music, da: physically-executed dance without music, im-m: imagined dance with music, im: imagined dance without music

^b^ χ2 with with degrees of freedom of Friedman-test, r-value effect size of the Friedman-test, z-value of the Friedman-test.

^c^Diff-rest 1: difference value from pre- to post-rest-measurement of the left test condition from test condition pair, Diff-rest 2: difference value from pre- to post-rest-measurement of the right test condition from test condition pair.
